# Anatomical Variation of the Tibia – a Principal Component Analysis

**DOI:** 10.1038/s41598-019-44092-8

**Published:** 2019-05-21

**Authors:** Liselore Quintens, Michiel Herteleer, Sanne Vancleef, Yannick Carette, Joost Duflou, Stefaan Nijs, Jos Vander Sloten, Harm Hoekstra

**Affiliations:** 1 KU Leuven - University of Leuven, Faculty of Medicine, Leuven, Belgium; 2 Department of Trauma Surgery, University Hospitals Leuven, Leuven, Belgium; 3 Biomedical Science Group, Organ Systems, KU Leuven – University of Leuven, Leuven, Belgium; 4 Department of Mechanical Engineering, KU Leuven - University of Leuven, Leuven, Belgium; 5 Department of Development and Regeneration, KU Leuven - University of Leuven, Leuven, Belgium

**Keywords:** Biomedical engineering, Bone

## Abstract

Conventional anatomically contoured plates do not adequately fit most tibiae. This emphasizes the need for a more thorough morphological study. Statistical shape models are promising tools to display anatomical variations within a population. Herein, we aim to provide a better insight into the anatomical variations of the tibia and tibia plateau. Seventy-nine CT scans of tibiae were segmented, and a principal component analysis was performed. Five morphologically important parameters were measured on the 3D models of the mean tibial shapes as well as the −3SD and +3 SD tibial shapes of the first five components. Longer, wider tibiae are related to a more rounded course of the posterior column, a less prominent tip of the medial malleolus, and a more posteriorly directed fibular notch. Varus/valgus deformations and the angulation of the posterior tibia plateau represent only a small percentage of the total variation. Right and left tibiae are not always perfectly symmetrical, especially not at the level of the tibia plateau. The largest degree of anatomical variation of the tibia is found in its length and around the tibia plateau. Because of the large variation in the anatomy, a more patient-specific approach could improve implant fit, anatomical reduction, biomechanical stability and hardware-related complications.

## Introduction

Tibial plateau fractures have a broad spectrum of morphological appearances and many classification systems have been proposed to describe this variability^1–3^. Fractures involving the posterior plateau are increasingly recognized as an important prognostic factor towards the functional outcome^4–6^. Therefore, there is a growing awareness of the need to address these posterior tibial plateau fractures, as unfixated posterior column fractures and subsequent sagittal malalignment predispose to significantly poorer patient-reported outcome scores^5^. As a consequence, there is an ongoing search for new operation strategies with the development of new implants^7–10^. Although several different approaches of the posterior column have been described^11^, the design of new implants is limited by the exposure of the posterior aspect of the tibia necessary in order to be able to perform an adequate reduction and fixation. Moreover, the design is complicated by anatomical variability of the tibia, wherein tibia exhibits more size-independent variability than other limb segments^12^. In contrast, adequate fit of these implants is of great importance on the one hand for both the biomechanical stability and to minimize soft-tissue irritation, and on the other hand, precontoured (anatomical) plates can serve as a template which facilitates the fracture reduction^13,14^. A good description of the anatomical variation that is present in these limb segments will help to increase our understanding of these complex anatomical regions. The goal of our study is not to assess how current implants fit on a statistical shape model (SSM) but to describe the anatomical variation that is present in certain anatomical features in order to reasonably describe more and less varying anatomical regions in the tibia. Other studies that used SSM focus on the quality of the models or described implant fit but did not describe the basic anatomical features that change throughout the different principal components^15–17^.

Currently, medical image-based population analysis is transforming the way standard implants are developed today. Virtual 3D models of the patient population allow for a much deeper insight in anatomical variation. In an era of personalized medicine, this allows us to strive for the best implant fit for every patient. Statistical shape models (SSM) are promising tools for displaying these variations within a population^18^. Therefore, we aimed to provide a better insight into the anatomical variations of the tibia and particularly the tibial plateau by analyzing the components of the SSM that we created.

## Materials and Methods

This study was completed in accordance with Belgian national legislation, research on cadavers is not within the scope of Belgian law regarding research on human subjects dd 7/5/2004. Hence the research was performed according to the Privacy law dd 8/12/1992.

### Data collection

79 Bilateral total tibiae from a forensic medical CT scan database were included in the study. The average age of the included patients was 50.6 ( ± 17.9 SD) years old, we included a total of 42 men and 37 women. The average length was 168.8 mm (±10.2 mm SD) and the average weight was 71.0 (±19.0 SD) kilograms. All included patients had the Belgian nationality. Exclusion was based on the presence of fractures or any other pathologic signs on the CT scans. The CT scans were made using a Siemens SOMATOM Definition Flash CT scanner. The acquisition parameters were the following: tube voltage 120 kVp, X-ray tube current 480 mA, slice thickness 1 mm, a maxtrix size of 512 × 512 pixels and a pixel size of 1.52344 mm. The bilateral total tibiae were segmented using Mimics Innovation Suite 19.0 (Materialise, Belgium). The tibias were segmented using the CT Bone Segmentation tool available in Mimics 19. A point on the cortex of the tibia was selected with a seed threshold of −389 and 0 sensitivity. The mask threshold was set at 226–3056 HU as finishing parameters a gap closing distance of 2 pixels was selected and long bones were filled. The part was calculated from the mask using the High Quality protocol which involves 2 smoothing iterations with a smoothing factor of 0.5. It also involves a triangle reduction using the advanced edge method (Tolerance 0.05 mm, Edge Angle 10 mm, 10 Iterations).

Reconstructed 3D tibiae were aligned according to the anatomical axis as proposed by the International Society of Biomechanics^19^. First, 4 landmarks were determined: Tip of the medial malleolus (MM), tip of the lateral malleolus (LM), the most medial point on the border of the medial tibial condyle (MC), and the most lateral point on the border of the lateral tibial condyle (LC). Next, the inter-malleolar point was determined, midway between MM and LM. The intercondylar point was determined midway between the MC and LC. Finally, the axis was determined as the line crossing both the inter-malleolar and the intercondylar point.

The resulting 3D meshes were imported in 3-matic (Materialise, Belgium) and optimized as follows. First, a wrap operation with a gap closing distance of 5.0 mm, a resulting offset of 0.35 mm and a smallest detail of 0.5 mm, was performed. Secondly, an adaptive remesh operation was performed, with a target triangle edge length of 1.8 mm and with preservation of the surface contours. Finally, the mesh was smoothed with a smoothing factor of 0.7 to obtain good quality meshes.

### Principal component analysis

To perform a PCA, corresponding meshes are required. Mesh correspondence was achieved by deforming one selected source tibia (the longest tibia) to every other target tibia, through non-rigid registration. The source was registered to the target bones. During our non-rigid registration, the source is first rigidly registered to the target. However, all target bones are not necessarily located at the same place in space. In order to perform PCA, all target bones should be aligned and therefore an extra alignment of all the registered target bones to the source, was performed before PCA. The iterative registration framework (Non Rigid Registration) was based on Danckaers *et al*.^20^ and was implemented in MATLAB (MathWorks, Natick, MA, USA). During surface registration, the source mesh is deformed to a target mesh. Firstly, the source is rigidly registered to the target and scaled. Then, iterative non-rigid registration is performed. Each iteration, firstly, corresponding points are identified. This is achieved by ray tracing: a ray is created along each source’s vertex normal and its eventual intersection point with the target geometry is calculated. The angle between the source’s vertex normal and the target’s face normal is calculated. If this angle is smaller than 45 degrees, the surface orientation at the two points is considered to be similar and the points corresponding. Thirdly, these corresponding points are input for the N-ICP-T registration algorithm described by Amberg *et al*.^18,21^. After performing the non-rigid registration, all tibiae were aligned using a Procrustes analysis and PCA was performed in Matlab (Mathworks, USA). In PCA, the mean tibial shape and its modes of variation or principal components (PCs) were calculated. The general formula for an SSM is

$$y=\bar{x}+\sum _{i=1}^{n}{w}_{i}{p}_{i}$$

In which y is a random tibia, x is the mean tibia mesh, w corresponds to the weighting factor and p denotes the mode of variation or PC.

An important property of these PCs is that shape variations present in each component are independent from the shape variations in the other components. The anatomical description was performed for the first five PCs. Firstly, the standard deviation was calculated for each component. Secondly, to visualize the influence of one particular PC on the mean clavicle, point clouds were generated from the SSM for which only that particular PC was given a weighting factor of −3SD (standard deviation) and +3 SD. To describe the PCs, point clouds of the mean clavicle and each mode of variation (−3SD and 3 SD) were imported into 3-matic and meshed using the automatic mesh settings (Point Distribution: Uniform, Smooth turned off). Two measures were performed to assess the quality of the SSMs. Firstly, the compactness was evaluated, this corresponds to the number of PCs required to describe a fixed preset percentage of variation. Secondly, the generalization was calculated. This number expresses how well a random tibia can be described with the SSM, by calculating the average distance between the original tibia mesh and the mesh obtained by the SSM^18^.

### Description of anatomical parameters

**Figure 1:**
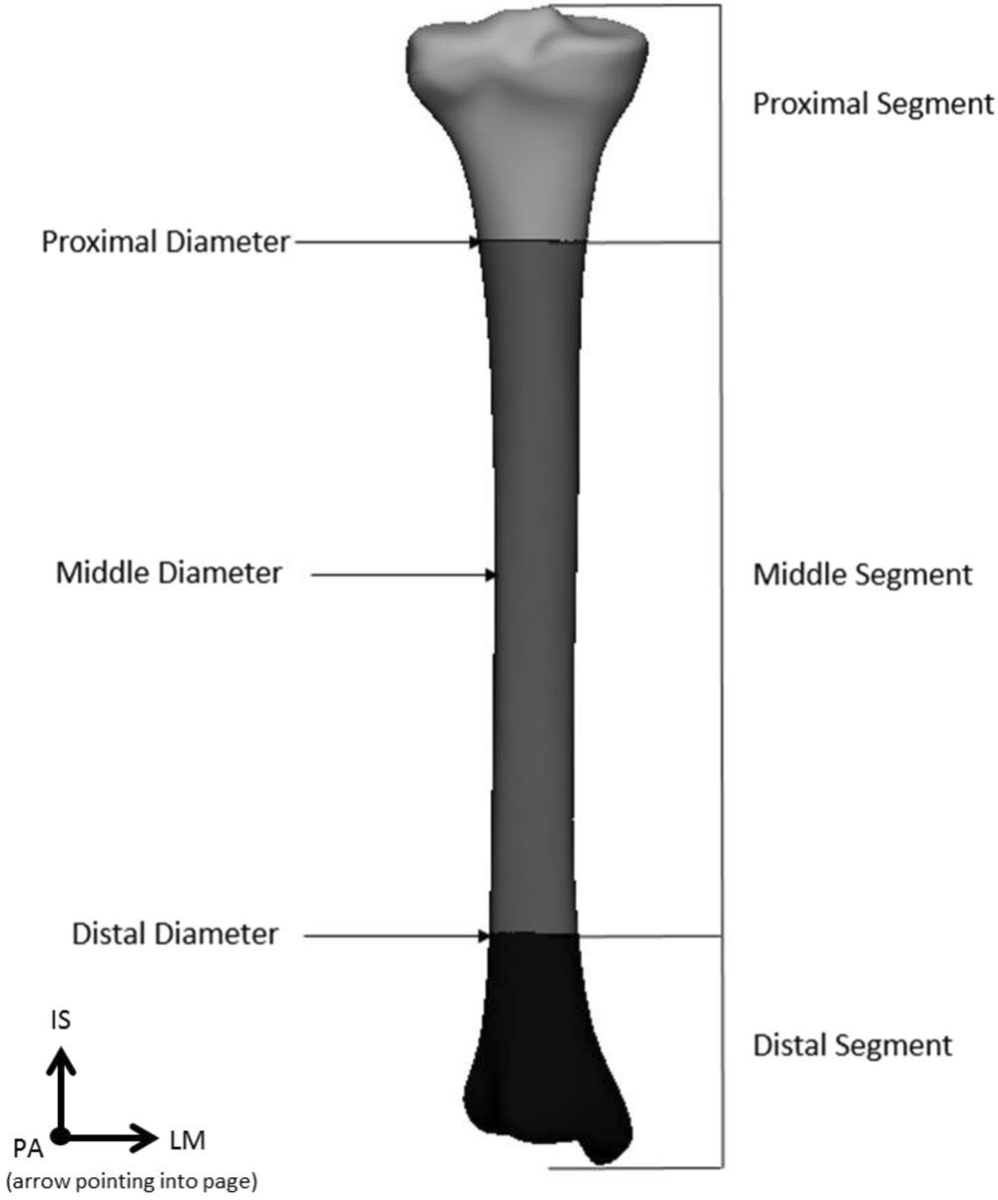
Measurement of length, volume and diameter. The measurements were carried out using the coordinate system as shown. The lateromedial (LM), posterior-anterior (PA) and inferior-superior (IS) directions are shown, with the IS direction corresponding with the anatomical axis.

**Figure 2:**
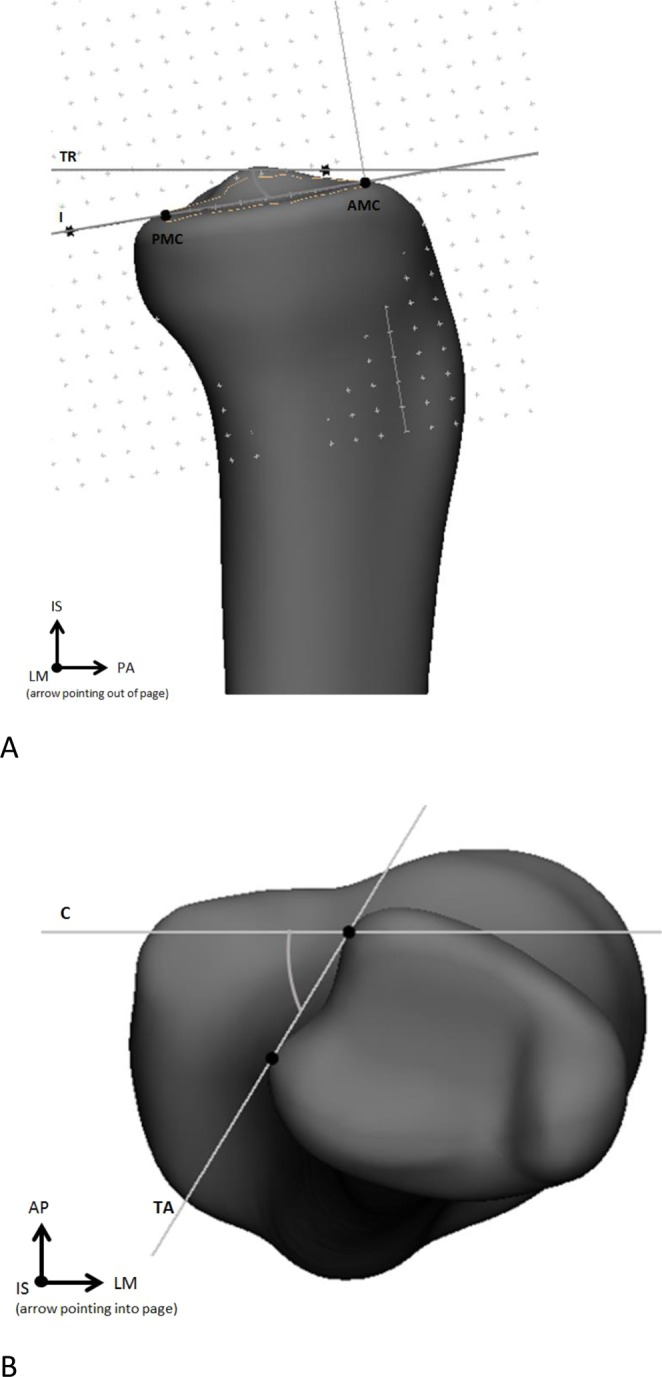
Measurement of (**A**) the slope of the tibia plateau and (**B**) the orientation of the fibular notch. The measurements were carried out using the coordinate system as shown. The lateromedial (LM), posterior-anterior (PA), anterior-posterior (AP) and inferior-superior (IS) directions are shown, with the IS direction corresponding to the anatomical axis. The transverse plane (TR), the inclination of the tibial plateau (I), the most anterior point on the border of the articular surface of the medial tibial condyle (AMC), the most posterior point on the border of the articular surface of the medial tibial condyle (PMC), the tangent plane (TA) and the coronal plane (C), all used in measuring the slope of the tibial plateau and the orientation of the fibular notch, are marked.

After meshing, a total of five morphologically important parameters (length, volume, diameter, slope of the tibial plateau, and orientation of the fibular notch) were measured from the mean tibial shape of the left and right 3D models, as well as the −3SD and +3SD tibial shapes of the first five components. First, the length was measured from the tip of the intercondylar eminentia to the tip of de medial malleolus, over the anatomical axis (as described above). Next, the tibia was divided into three segments by making transverse cross sections (cross sections perpendicular to the anatomical axis) at 20% and 80% of the tibial lengths, as shown in Fig. 1. This allowed 3-matic to determine the volumes of the proximal, middle and distal segments individually. A third transverse cross section was made at 50%. At each cross section, a 2D outline was made of the contour of the tibia crossing these transverse sections. Using these 2D outlines of the tibial contour, the largest diameter at 20%, 50% and 80% of the tibias length were measured. Next, the slopes of the medial and lateral tibial plateaus were measured (Fig. 2A). The slope of the tibia was defined as the angle between the transverse plane (a perpendicular plane to the axis of the tibia) on a sagittal image, and the inclination of the tibia plateau^22^. The inclination of the medial tibial plateau was determined as the line connecting the most anterior point on the border of the articular surface of the medial tibial condyle (AMC), and the most posterior point on the border of the articular surface of the medial tibial condyle (PMC). The same was done for the lateral tibia plateau. Lastly, the orientations of the fibular notch were determined. This was done by fitting a tangent plane for every tibia so that it would cover the fibular notches, perpendicular to the transverse plane. The orientations of the fibular notch were defined as the angles measured between these tangent planes and the coronal plane of the tibia.

## Results

### Evaluation of the statistical shape model

**Figure 3:**
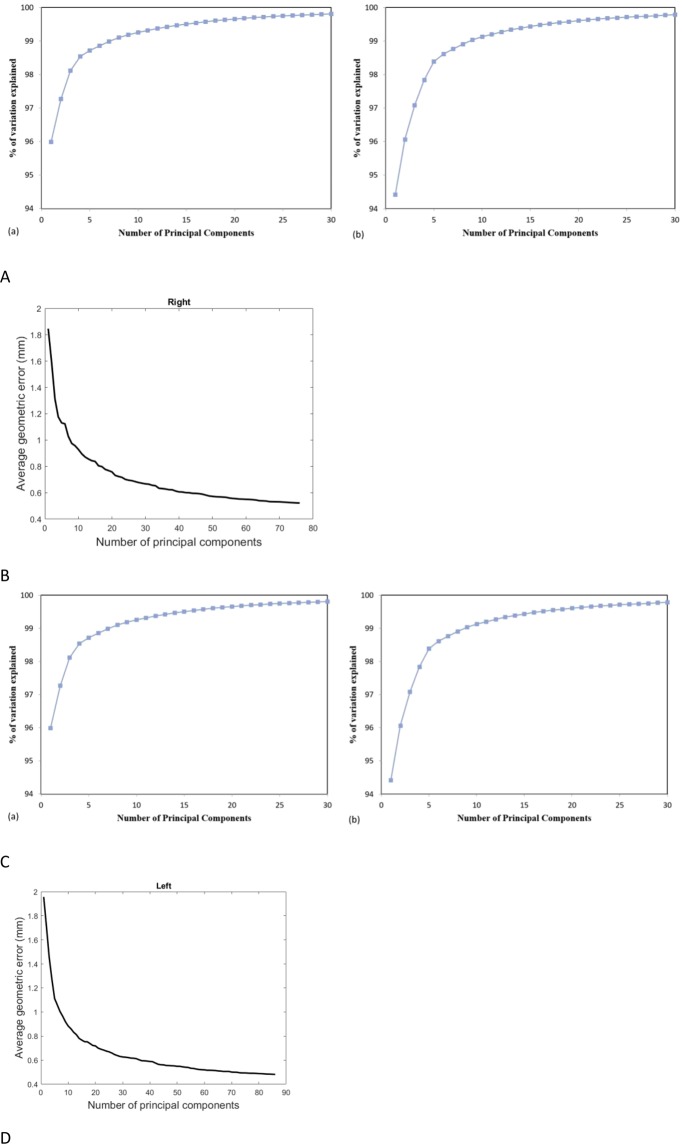
Percentage of the anatomical shape variation of the tibia explained by the number of principal components for the (**A**) right and (**C**) left tibia. Generalization graph of (**B**) right and (**D**) left tibiae.

The first five components of the right tibia accounted for 98.8% of the anatomical shape variation of the tibia. The first principal component accounted for 96.0%, the second accounted for 1.3%, the third accounted for 0.9%, the fourth accounted for 0.4% and the fifth component accounted for the remaining 0.2% of the variation (Fig. 3A). Generalization right (Fig. 3B) shows that a random right tibia can be described by the SSM of right tibiae, with an average accuracy of 0.522 mm.

The first five components of the left tibia account for 98.4% of the anatomical shape variation of the tibia. The first principal component accounts for 94.4%, the second accounts for 1.6%, the third accounts for 1.0%, the fourth accounts for 0.8% and the fifth component accounts for the remaining 0.6% of the variation (Fig. 3C). Generalization left (Fig. 3D) shows that a random left tibia can be described by the SSM of left tibiae, with an average accuracy of 0.481 mm.

The differences for every morphological parameter between the −3SD and +3 SD shapes for every principal component of the right and left tibia are shown in Table 1 and Table 2.

**Table 1:** Difference for every morphological parameter between the −3SD and +3 SD shapes for every principal component (PC) of the right tibia.

	PC1	PC2	PC3	PC4	PC5
Length	182 mm	1 mm	0 mm	1 mm	1 mm
Volume (tibia)	371.5 cm^3^	213.6 cm^3^	27.3 cm^3^	90.1 cm^3^	38.0 cm^3^
Volume (proximal segment)	156.2 cm^3^	97.4 cm^3^	17.5 cm^3^	34.6 cm^3^	2.7 cm^3^
Volume (mid segment)	148.4 cm^3^	78.0 cm^3^	0.6 cm^3^	40.2 cm^3^	35.9 cm^3^
Volume (distal segment)	66.9 cm^3^	38.3 cm^3^	9.2 cm^3^	15.3 cm^3^	0.5 cm^3^
Diameter (proximal)	12 mm	12 mm	3 mm	7 mm	4 mm
Diameter (middle)	10 mm	6 mm	3 mm	3 mm	4 mm
Diameter (distal)	5 mm	6 mm	2 mm	5 mm	3 mm
Slope (medial plateau)	2°	4°	11°	2°	2°
Slope (lateral plateau)	0°	3°	11°	2°	1°
Fibular notch orientation	9°	14°	7°	15°	25°

**Table 2:** Difference for every morphological parameter between the −3SD and +3 SD shapes for every principal component (PC) of the left tibia.

	PC1	PC2	PC3	PC4	PC5
Length	182 mm	1 mm	4 mm	7 mm	2 mm
Volume (tibia)	388.5 cm^3^	238.8 cm^3^	34.2 cm^3^	1 cm^3^	78.9 cm^3^
Volume (proximal segment)	157.9 cm^3^	105.5 cm^3^	18.9 cm^3^	−1.9 cm^3^	26.1 cm^3^
Volume (mid segment)	157.4 cm^3^	90.7 cm^3^	3.7 cm^3^	−1 cm^3^	41.3 cm^3^
Volume (distal segment)	73.1 cm^3^	42.6 cm^3^	11.6 cm^3^	3.5 cm^3^	11.5 cm^3^
Diameter (proximal)	12 mm	16 mm	5 mm	0 mm	5 mm
Diameter (middle)	11 mm	8 mm	3 mm	2 mm	4 mm
Diameter (distal)	5 mm	7 mm	2 mm	0 mm	3 mm
Slope (medial plateau)	2°	3°	11°	8°	0°
Slope (lateral plateau)	0°	1°	4°	5°	2°
Fibular notch orientation	9°	9°	15°	5°	10°

### Right-sided tibia

**Figure 4:**
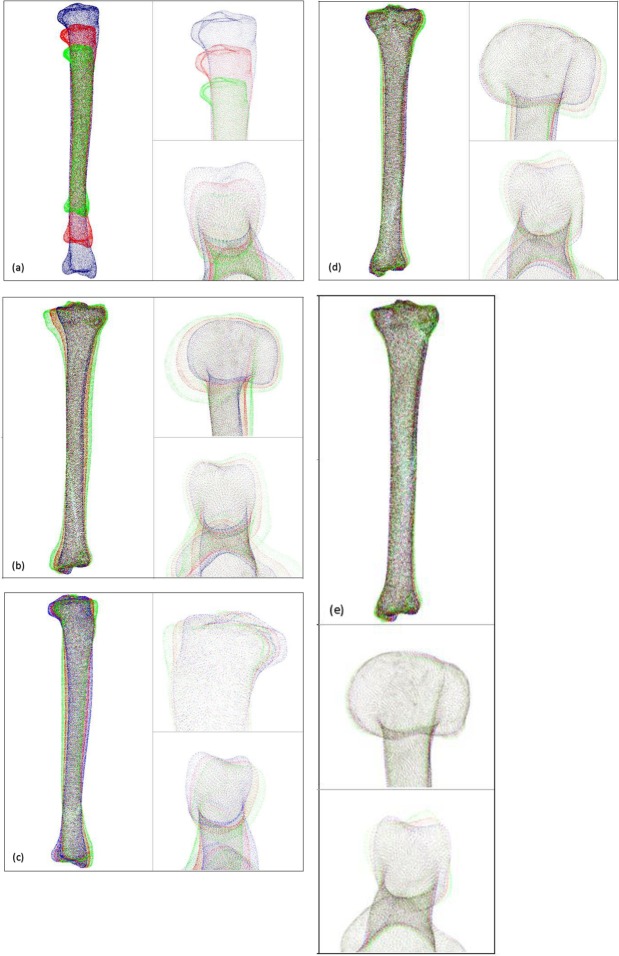
The first five principal components of the right tibia. For every principal component, 3D models of the mean tibial shape and the −3SD and +3 SD tibial shapes are displayed. A view of the complete tibia, as well as a magnified image of the proximal and distal end of the tibia, is shown for every principal component. (**a**) First principal component (PC). (**b**) Second PC. (**c**) Third PC. (**d**) Fourth PC. (**e**) Fifth PC.

The first principal component (Fig. 4a) reflects the variation of the length of the tibia as well as its variation in width. The length of the tibia varied from 286 mm (−3SD) to 468 mm (+3 SD) with an average length of 377 mm. The largest difference in diameter, up to 12 mm, was located at the proximal end of the tibia. Although the slopes of the medial and lateral tibial plateaus were not related to the length or width of the tibiae, the posterior columns of shorter tibiae showed a steeper and more angular course, whereas the posterior columns of longer tibiae showed a more rounded course. On the distal end, the tip of the medial malleolus was more prominent in shorter tibiae and the fibular notch was directed up to 9° more laterally.

The second principal component (Fig. 4b) primarily showed a relationship between valgus deformities of the tibia and the size of the medial tibial plateau. A valgus deformation of the tibial shaft was related to a smaller medial plateau compared with straighter tibiae of the same length. Valgus shaped, small tibiae also showed more posteriorly tilted plateaus and more a posteriorly oriented fibular notch. The difference in slope was slightly more prominent at the medial plateau.

The third principal component (Fig. 4c) showed that an angular shaped posterior tibial plateau is related to an increased anterior bowing of the shaft. The increase in the posterior tilt was unrelated to the volume or diameter of the tibia. Tibiae with an increased anterior bowing also showed a more posteriorly oriented fibular notch.

The fourth principal component (Fig. 4d) showed a relationship between valgus/varus deformities of the tibia and the size of the lateral plateau. A varus deformation of the tibial shaft was related to a smaller lateral plateau compared with straight tibiae of the same length. This relationship was less pronounced than that concerning the medial plateau described in the second principal component. The slope of the medial and lateral tibial plateau was not clearly related to the degree of varus malformation of the tibia. The fibular notch of more varus shaped tibiae was oriented up to 15° more posteriorly.

The fifth principal component (Fig. 4e) showed that the orientation of the fibular notch can vary independently of any other morphological structures.

### Left-sided tibia

The first two left principal components (Supplement Figure S1a,b) were not relevantly different from the first two right principal components. In the third principal component (Supplement Figure S1c) a slight distinction between right and left tibiae became apparent. Though the component also showed that a more posteriorly tilted posterior tibial plateau was related to an increased anterior bowing of the shaft, it was primarily the medial plateau that accounted for this relationship (11° respectively 4°). In relation to the difference in slope of the medial and lateral plateau, the more posterior orientation of the fibular notch in relation with an increased anterior bowing was more prominent compared with the right tibia (Δ15° respectively Δ7°).

The fourth and the fifth principal components markedly differed from the components of the right tibiae. The fourth principal component (Supplement Figure S1d) showed that the slope of the tibia can vary independently of any other morphological structure. The posterior column of tibiae with a more posterior tilted plateau had a more rounded course whereas the posterior columns of tibiae with a smaller slope had a steeper more angular course.

The fifth principal component (Supplement Figure S1e) showed a relationship between valgus/varus deformities of the tibia and the size of the lateral plateau. A varus deformation of the tibial shaft was related to a bigger lateral plateau and a more laterally directed fibular notch compared with straight tibiae of the same length.

## Discussion

### Clinical significance

In this study we aimed to evaluate anatomical variations of the tibia, particularly the tibia plateau, by creating a SSM, since adequate fit of new implants is essential for both the biomechanical stability of (posterior) tibial plateau fractures and protection of the underlying soft-tissues^13,14^. Furthermore, precontoured plates can serve better as a template in order to reduce the fracture fragments^23^. Defining anatomical variations of the tibia is of importance not just for surgeons, but also for researchers and commercial partners, both in applied and fundamental research.

The innovation of our analysis, however, lies in the description of the relationship between morphological features within each component. As expected, the largest variation in the tibia anatomy was found in its length. Longer, wider tibiae are related to a more rounded course of the posterior column, a less prominent tip of the medial malleolus and a more posteriorly directed fibular notch. Although the varus/valgus deformities, and the angulation of the posterior tibial plateau represent only a small percentage of the total variation, the variation within the different components is large and has a direct relationship with the morphology of the tibia plateau.

In computer-assisted reconstructive surgeries, the contralateral anatomy is established as the best available reconstruction template. However, this assumes no intra-individual bilateral differences. Radzi *et al*.^24^ already assessed bilateral geometrical differences of the tibia, using both 2D and 3D measurements in various anatomical regions. Most of the 2D measurements showed significant differences, except for the lateral plateau and distal subchondral bone surface measurements. Also the 3D measurements showed differences between the left and right tibia, for the full tibia, proximal tibia, mid diaphyseal region and medial tibia plateau. This shows that there is indeed a difference between bilateral tibiae, especially at the level of the proximal tibia and medial tibial plateau, justifying more thorough morphological studies. However, it should be noted that the clinical relevance of these differences, though significant, are disputable, since they are only in the range of less than 1 mm. Although a symmetry in morphometric dimensions between right and left tibiae was assumed^25^, our results showed that there is, however, a difference in shape variation between the right and the left tibiae in the fourth and fifth component. The nearly symmetrical shape variation in the first two components, accounting for 97.3% of the anatomical shape variation, which supports the assumption of a good correlation in the morphometric dimensions of right and left tibiae. However, the results of our analysis suggest that the tibia is not perfectly symmetrical, and this particularly applies to the tibia plateau.

### Advantages of PCA and SSM for posterior tibial plateau fractures

SSM and PCA are commonly used analysis methods but it is only recently that they have been applied to the field of anatomy. Here we have shown how SSM allow for analysis of complex free-shape such as the tibia. Subsequently, this approach enables us to correlate shape variations within each component and therefore gives us a more thorough description of the shape variations present in the population.

**Figure 5:**
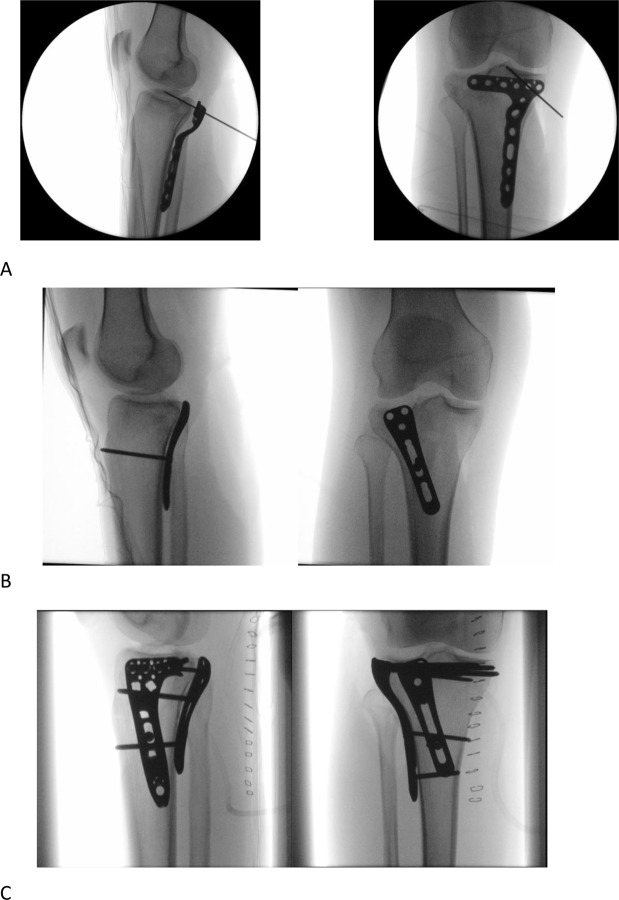
A two-column tibial plateau fracture according to the revised three-column classification approach^31^. (**A**) inadequate posterior buttress using the WAVE proximal posterior tibia plate (7S-Medical, Oberkirch Switzerland) as shown in the sagittal plane (left image), due to a less angular shaped posterior tibial plateau (which is related to anterior bowing in the 3^rd^ PC). (**B**) therefore, it was decided to apply a less angulated locking compression plate (DePuy Synthes); left = sagittal plane, right = coronal plane. (**C**), final osteosynthesis of the two column tibial plateau fracture, using a posterior and lateral plate displayed in 2 directions.

The anatomical variation presented in this study explains why currently existing anatomical plates don’t fit sufficiently (Fig. 5). As the shape of the tibial plateau varies largely throughout the first five components it means that a trauma or orthopaedic surgeon would need 243 (three shapes in each component, to the power of five, because of the five principal components that need to be included) differently shaped plates to choose from, to have an adequately fitting plate in 98.8% of the population. Although this number can be reduced by choosing a zone on the tibia that exhibits less variation, which was also the conclusion of Kozic *et al*.^17^ who investigated this for the lateral tibia, however they did not evaluatie this for the posterior and medial tibia plateau, which are of equal importance in treating complex tibial plateau fractures. In the nearby future, surgeons will be able to plan preoperatively complex fractures, such as tibial plateau fractures, with the help of statistical shape models. These statistical shape models can be used in a 3D environment to predict the original shape of the tibia and re-align the fracture fragments in the original position^25^. Based on this 3D reconstruction, the necessary implants can be chosen and selected or even designed beforehand. This could reduce operating time, improve anatomic reduction and minimize soft-tissue irritation. Pre-operative individualized planning, in combination with implants that are designed with the help of statistical shape models, could result in a large step forward in the treatment of complex fractures. SSM are increasingly performed in orthopedic research. A recent study by Vlachopoulos *et al*. for example also showed that a SSM is an accurate tool to predict the patient-specific anatomy of the proximal and distal aspects of the humerus^26^. Although this was not the focus of our study our SSM could be used for this as well. Since rising evidence on intra-individual bilateral differences, accurately predicting the pre traumatic anatomy of the bone from the posttraumatic condition can be an interesting development in orthopedic research and is of fundamental importance for computer-assisted reconstructive surgeries.

Due to a large anatomical variability of the tibia, conventional precontoured plates often don’t adequately fit. As consequence, inadequate reductions and/or fixation can lead to malalignment and incongruity of the joint surface, and biomechanical and functional problems^23,27,28^. Correctly addressing tibial fractures is therefore increasingly accepted as an important prognostic factor for functional outcome^5^. We believe that a lack of properly precontoured plating systems can partially explain the modest results of, for example, tibial plateau fracture treatment^29–32^. The results of our study show that there is a large anatomical variation of the tibia plateau. Therefore, it is unlikely that an of the shelf implant can fit all these possible shape variations. We look forward towards future clinical trials investigating the clinical benefit of better fitting of custom made implants, since for sure there is an anatomical need.

### Limitations of the study

The most important limitation of our study is that due to the anonymized patient data we could not relate any of the information regarding the shape to other patients characteristics such as patients sex, length, ethnicity or age. However, Tümer *et al*.^15^ investigated shape variations and symmetry in the lower limb and concluded that gender does not explain shape variations in the bone of the lower limb. They also concluded that in general a symmetry between the left and right tibia cannot be assumed and the contralateral side cannot always be used as a template for planning an arthroplasty surgery or a corrective osteotomy. In contrast, statistical shape models can be used to predict the shape of a certain bone based on a part of the bone^33,34^. Although this type of analysis was not part of our research we suggest this as a further prospect for researchers.

## Supplementary information

Supplement Figure 5

## Data Availability

The datasets generated during and/or analysed during the current study are available from the corresponding author on reasonable request.
