# Supplementary material for: Anatomical Variation of the Tibia – a Principal Component Analysis
Source: Sci Rep. 2019 May 21;9:7649. doi: 10.1038/s41598-019-44092-8 (PMC6529455; doi:10.1038/s41598-019-44092-8)

Anatomical Variation of the Tibia – a Principal Component Analysis

Liselore Quintens[1*], Michiel Herteleer[2,3*], Sanne Vancleef[4], Yannick Carette[4], Joost Duflou[4], Stefaan Nijs[2,5], Jos Vander Sloten[4], Harm Hoekstra[2,5]

[*]Both authors equally contributed to the research paper

[1] KU Leuven - University of Leuven, Faculty of Medicine, Leuven, Belgium

[2] Department of Trauma Surgery, University Hospitals Leuven, Leuven, Belgium

[3] Biomedical Science Group, Organ Systems, KU Leuven – University of Leuven, Leuven, Belgium

[4] Department of Mechanical Engineering, KU Leuven - University of Leuven, Leuven, Belgium

[5] Department of Development and Regeneration, KU Leuven - University of Leuven, Leuven, Belgium

Figure S1: The first five principal components of the left tibia. For every principal component, 3D models of the mean tibial shape and the -3SD and +3SD tibial shapes are displayed. A view of the complete tibia, as well as a magnified image of the proximal and distal end of the tibia, is shown for every principal component. (a) First principal component (PC). (b) Second PC. (c) Third PC. (d) Fourth PC. (e) Fifth PC.

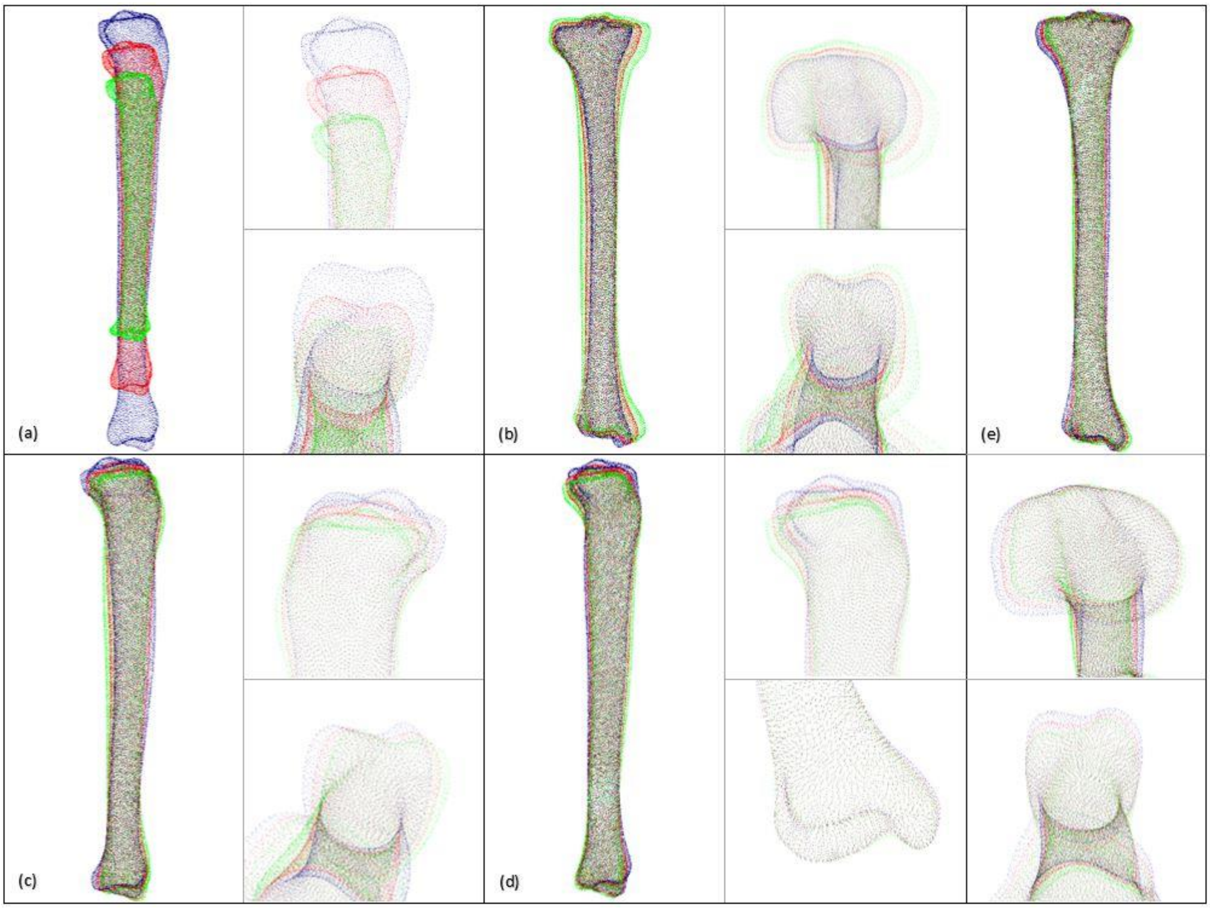

Supplement: Supplementary file 1 — Supplement Figure 5 [file 41598_2019_44092_MOESM1_ESM.pdf]
